# Impact of Out-of-pocket Costs on Varenicline Utilization and Persistence

**DOI:** 10.36469/9888

**Published:** 2014-08-06

**Authors:** Aaron Galaznik, Katherine Cappell, Leslie Montejano, Geoffrey Makinson, Kelly H. Zou, Gregory Lenhart

**Affiliations:** 1 Pfizer Inc., New York, NY, USA; 2 Truven Health Analytics, Ann Arbor, MI, USA

**Keywords:** varenicline, utilization, smoking cessation, out-of-pocket

## Abstract

**Background:** Varenicline is a smoking cessation medication.

**Objectives:** We analyzed patients’ out-of-pocket costs and utilization of and persistence with varenicline.

**Methods:** De-identified claims data in the MarketScan® Commercial Claims and Encounters Database were analyzed retrospectively. Participants were all patients at least 18 years of age continuously enrolled in plans during 2009. Plans were categorized according to restriction (no coverage; prior authorization; smoking cessation program requirement; no restrictions) and out-of-pocket cost for a 30-day supply (low: <US$12; medium: US$12–24.99; high: ≥US$25). The main outcome measures were utilization (defined as presence of a drug claim) and persistence (according to days’ supply and number of days to discontinuation). Generalized linear models and time-to-event analyses were conducted.

**Results:** There were 142,251, 458,966 and 222,241 individuals in the low, medium and high out-of-pocket cohorts, respectively. The reference group for all comparisons was the cohort with no access restrictions and low out-of-pocket costs. Higher out-of-pocket cost was associated with a lower likelihood of varenicline initiation for both the prior authorization (odds ratio [OR]=0.10, p<0.001) and smoking cessation program requirement (OR=0.19, p<0.001) groups, versus the no restriction cohort. Within the no access restriction cohort, subjects in the high out-of-pocket group were half as likely to complete a varenicline course versus the low out-of-pocket group (OR=0.47; p<0.002). Conversely, for the smoking cessation program requirement cohort, compared to the low out-of-pocket no restriction cohort, subjects who were in the high out-of-pocket group were more likely to complete a varenicline course (OR=0.70; p=0.13) than those in the low out-of-pocket group (OR=0.38; p=0.04).

**Conclusions:** Higher varenicline out-of-pocket costs were generally associated with lower utilization of and persistence with treatment. These findings have implications for coverage policies in health plans and employers seeking to encourage smoking cessation.

## BACKGROUND

Smokers have lower productivity, higher rates of absenteeism, and more workplace accidents and injuries compared with non-smokers. Furthermore, smokers incur healthcare costs that are 20–50% higher than those of non-smokers.[1-3] Despite the known adverse health and economic consequences associated with smoking, tobacco use remains the leading cause of preventable death and disease in the United States, claiming nearly 450,000 lives annually.[4] An estimated 70% of smokers express the desire to quit, and although 40–50% of these are known to have made at least one quit attempt, only 6% are successful.[5-7]

Several effective pharmacotherapies are currently licensed for use as smoking cessation aids, and include nicotine replacement therapy, sustained-release bupropion and varenicline. Although clinical treatment guidelines recommend the use of smoking cessation pharmacotherapy,[8] and evidence suggests that such action significantly increases the likelihood of quitting, the majority of smokers who make a quit attempt do not seek assistance.[6, 9,10] This may be in part due to drug utilization management techniques that can demand high copayments, thus reducing smokers' motivation to make use of such treatment.[11] There is growing literature on the impact of utilization management strategies on the use of medications. Higher copayments have been shown to be associated with lower rates of treatment initiation[12] and medication adherence,[13,14] and conversely, reducing copayments has been shown to improve patient adherence to therapy.[15] A study examining the effect of copayments among smokers taking varenicline revealed that those with a low (US$0–5) copayment were more likely to fill a prescription for any smoking medication compared with those who had a high (≥US$31) copayment.[16] Another study reported that higher copayments were associated with failure to fill a varenicline prescription.[17]

Results of a number of meta-analyses examining the comparative effectiveness of smoking cessation medications have confirmed that smokers taking varenicline are more successful at quitting and remaining abstinent compared with those using nicotine replacement therapy or sustained-release bupropion.[8,10,18] Furthermore, cost-benefit analyses found varenicline to be the most cost-effective pharmacotherapy for treating tobacco dependence.[19-22] However, despite these results, varenicline is the most frequently targeted smoking cessation pharmacotherapy for access restrictions.[16,23] Our study was conducted to explore the relationship between patient out-of-pocket costs and the utilization of and persistence with varenicline treatment.

## METHODS

### Study Design

This retrospective, observational cohort study was conducted using data from the MarketScan® Commercial Claims and Encounters Database, which is a database containing inpatient, outpatient and prescription drug claims from employees and dependents who receive health insurance coverage from a group of approximately 100 self-insured companies in the United States. Our study, which assessed the relationship between out-of-pocket costs and utilization of and persistence with varenicline, was conducted as part of a study that assessed access restrictions. Therefore, to assess the impact of access restrictions on varenicline use, employer health plans with available documentation on varenicline access restrictions in 2009 were selected and initially categorized into four cohorts according to type of restriction: (1) no coverage provided; (2) prior authorization required for coverage; (3) participation in a smoking cessation program required for coverage; and (4) no coverage restrictions. Results of this analysis are reported elsewhere.[24] Healthcare claims for varenicline were not available from plans that did not provide coverage and consequently, there were no out-of-pocket costs to be measured from these claims. Therefore plans with no coverage were excluded from this analysis on out-of-pocket costs. Using observed mean out-of-pocket costs for varenicline in each health plan, the three remaining cohorts were further categorized according to out-of-pocket costs for a 30-day supply of varenicline as (1) low (<US$12), (2) medium (US$12–24.99), and (3) high (≥US$25). To ensure a reliable estimate of a health plan's out-of-pocket costs, health plans with fewer than 100 varenicline users were excluded. The cut points for the three out-of-pocket cohorts were selected from the distribution of mean out-of-pocket costs, with roughly one-third of patients falling into each out-of-pocket cohort.

### Study Sample

The study sample included patients aged ≥18 years who were enrolled in selected health plans from 1 January to 31 December 2009. Individuals who were not continuously enrolled in health plans with prescription drug coverage during the calendar years 2008 (pre-period) and 2009 (study period) were excluded. Because data from the first quarter of 2010 were required to assess persistence for patients initiating varenicline treatment at the end of 2009, only those patients who were also continuously enrolled in health plans from January 2010 to March 2010 were included in the persistence analyses.

### Medication Utilization and Persistence

Measures of varenicline utilization and persistence were obtained from analysis of outpatient pharmacy claims. Utilization was assessed for all patients in the sample and was defined as the presence of a drug claim by a plan member. Persistence was measured for patients who initiated varenicline in 2009 according to: days' supply of varenicline across all claims incurred in 2009, number of days to discontinuation and whether patients completed a full course of therapy. Patients with claims for varenicline in 2009 were considered to have initiated varenicline therapy in 2009 unless they also had varenicline claims in the last quarter of 2008. Patients who initiated varenicline in 2009 and who had at least 90 days' supply of the medication within 113 days of the earliest fill date in 2009 were considered to have undergone a complete course of varenicline therapy. A 113-day window was selected to allow for possible delays in medication initiation after the initial prescription fill, as well as for brief gaps between fills.[25]

### Patient Demographics

Information on patient demographics was obtained from enrollment records and was measured on January 1, 2009. Pre-index healthcare utilization and comorbidities were derived from claims incurred in the calendar year 2008. General comorbid burden was measured from non-diagnostic (i.e. non-laboratory and radiology) claims using the Deyo–Charlson comorbidity index, with higher scores indicating higher comorbid burden.[26]

### Statistical Analysis

Differences between categorical variables were assessed using the chi-squared test and those for continuous variables were assessed using a two-sided Student's t-test. A two-sided p-value <0.05 was considered statistically significant. The odds ratios of varenicline use were computed using logistic regression to control for differences between cohorts. Persistence on varenicline was assessed by modeling days' supply using a generalized linear model with negative binomial error distribution and log link. In addition, time to discontinuation of varenicline was assessed using Cox's proportional hazards model in a time-to-event analysis. As both the type of access restriction and the level of out-of-pocket cost could have impacted varenicline use and persistence, both were included as explanatory variables in the models. For use in the models, the out-of-pocket cost groups were collapsed to two categories: low (<US$25) and high (≥US$25).

Because the samples comprised very few patients aged >65 years, this age group was excluded from the models analysis. The demographic characteristics controlled for in the models included age, sex, population density (urban vs. rural), geographic region, median household income in the same zip code as the primary insured and percent of college graduates in the same zip code as the primary insured. Clinical characteristics controlled for in the models included prior use of varenicline or nicotine replacement therapy, prior physician office visit, Deyo–Charlson comorbidity index score, tobacco abuse/dependence and the presence of specific comorbidities (listed in Table 1).

## RESULTS

### Study Participants

The study sample was selected from among individuals continuously enrolled in the health plans selected for the analysis from 2008 to 2009 (N=1,341,519). After excluding individuals who were (1) enrolled in plans that did not cover varenicline (n=454,419), and (2) those who were enrolled in plans for which the out-of-pocket payment amount for varenicline could not be reliably determined (n=63,642), a total of 823,458 patients met the study inclusion criteria. The study sample included 142,251 individuals in the low out-of-pocket cohort, 458,966 individuals in the medium out-of-pocket cohort and 222,241 individuals in the high out-of-pocket cohort. The low out-of-pocket and medium out-of-pocket groups were largely composed of individuals in plans with no access restrictions on varenicline, whereas the high out-of-pocket group was mainly composed of people in plans that required prior authorization or enrollment in a smoking cessation program. The demographic and clinical characteristics of the patients included in the out-of-pocket cost analyses are presented in Table 1.

### Medication Utilization

In 2009, the proportion of patients filling at least one prescription for varenicline was higher among patients within the low (2.8%; p<0.0001 vs. high costs) and medium (1.9%; p<0.0001 vs. high costs) out-of-pocket costs cohorts, than the proportion among patients in the high out-of-pocket costs cohort (0.4%).

Table 2 shows the impact of access restriction and level of cost-sharing on the probability of varenicline treatment, based on logistic regression analysis. The reference group for all comparisons was the cohort with no access restrictions and low out-of-pocket costs. Among patients with no access restrictions on the use of varenicline, the odds of varenicline treatment were 14% lower in the high out-of-pocket cohort than the low out-of-pocket cohort (OR=0.86; p=0.006). The odds of varenicline treatment were also significantly lower in most other groups, including prior authorization and low out-of-pocket (59% decrease; OR=0.41; p<0.001) or high out-of-pocket costs (90% decrease; OR=0.10; p<0.001), and smoking cessation program requirement and high out-of-pocket costs (81% decrease; OR=0.19; p<0.001), compared with no access restrictions and low out-of-pocket costs. The only exception was that the odds of varenicline treatment was significantly higher (37% increase; OR=1.37; p<0.001) with a smoking cessation program requirement and low out-of-pocket costs compared with the no restriction, low out-of-pocket costs (reference) group.

**Table 1.** Demographic and Pre-index Clinical Characteristics

| Subject Characteristics | Varenicline Out-of-pocket Cost ≤US$11.99 | Varenicline Out-of-pocket Cost US$12–24.99 | Varenicline Out-of-pocket Cost ≥US$25 |
|---|---|---|---|
| | **N = 142,251** | **N = 458,966** | **N = 222,241** |
| Mean age, years (SD) | 44.6 (12.7) | 49.5 (13.1) | 43.7 (12.3) |
| Male, n (%) | 57,147 (40.2) | 207,609 (45.2) | 92,634 (41.7) |
| Urban (vs. rural) residence, n (%) | 97,147 (68.3) | 355,821 (77.5) | 110,095 (49.5) |
| **Geographic Region, n (%)** | | | |
| Northeast | 3,419 (2.4) | 20,979 (4.6) | 1,588 (0.7) |
| North Central | 6,176 (4.3) | 259,391 (56.5) | 30,152 (13.6) |
| South | 128,092 (90.0) | 172,608 (37.6) | 185,943 (83.7) |
| West | 4,546 (3.2) | 5,824 (1.3) | 4,410 (2.0) |
| Other/Unknown | 18 (0) | 164 (0) | 148 (0.1) |
| Median household income*, US$ (range) | 35,718 (8,882–152,338) | 43,091 (2,499–200,001) | 31,691 (5,000–200,000) |
| Deyo–Charlson comorbidity index (SD) | 0.3 (0.8) | 0.4 (0.9) | 0.2 (0.7) |
| Tobacco abuse/dependence, n (%) | 1,426 (1.00) | 5,673 (1.24) | 1,547 (0.70) |
| **Comorbid Conditions, n (%)** | | | |
| Allergic rhinitis | 9,383 (6.60) | 21,299 (4.64) | 11,051 (4.97) |
| Asthma | 3,761 (2.64) | 14,961 (3.26) | 4,160 (1.87) |
| Bronchitis, acute or chronic | 3,014 (2.12) | 10,250 (2.23) | 5,214 (2.35) |
| Stroke/TIA | 1,098 (0.77) | 6,348 (1.38) | 1,134 (0.51) |
| COPD | 1,145 (0.80) | 7,520 (1.64) | 1,114 (0.50) |
| Coronary artery disease | 4,202 (2.95) | 22,776 (4.96) | 4,341 (1.95) |
| Depression | 5,150 (3.62) | 21,475 (4.68) | 5,117 (2.30) |
| Diabetes | 10,432 (7.33) | 42,811 (9.33) | 14,010 (6.30) |
| Dyslipidemia | 13,697 (9.63) | 52,023 (11.33) | 12,422 (5.59) |
| Emphysema | 162 (0.11) | 854 (0.19) | 112 (0.05) |
| Hypertension | 25,773 (18.12) | 96,225 (20.97) | 37,242 (16.76) |
| Lung/bronchial cancer | 75 (0.05) | 533 (0.12) | 113 (0.05) |
| Osteoporosis | 908 (0.64) | 3,152 (0.69) | 710 (0.32) |
| Peripheral vascular disease | 472 (0.33) | 2,787 (0.61) | 519 (0.23) |
| Pneumonia | 1,568 (1.10) | 5,702 (1.24) | 2,148 (0.97) |
| Sinusitis, acute or chronic | 23,080 (16.22) | 48,942 (10.66) | 26,671 (12.00) |
| Ulcer | 301 (0.21) | 1,258 (0.27) | 507 (0.23) |
| **Healthcare utilization, n (%)** | | | |
| Physician office visits | 123,650 (86.92) | 381,229 (83.06) | 166,391 (74.87) |
| Varenicline | 2,727 (1.92) | 10,413 (2.27) | 1,187 (0.53) |
| Nicotine replacement therapy | 41 (0.03) | 598 (0.13) | 16 (0.01) |

*Measured at the 5-digit zip code level.

COPD: chronic obstructive pulmonary disease; SD: standard deviation; TIA: transient ischemic attack

Baseline characteristics were compared between all groups using the chi-squared test and Student's t-test. Out-of-pocket cost ≥$25 was compared to out-of-pocket cost $12-$24.99; and out-of-pocket cost ≥$25 was compared to out-of-pocket cost ≤$11.99. No adjustment was made for multiple comparisons. There were significant differences (all $p<0.05$) between cohorts in all demographic characteristics measured.

**Table 2.** Impact of Access Restrictions and Out-of-pocket Cost of Varenicline Utilization

| Outcome | Access Level and Patient Cost | | N | Odds Ratio | 95% CI |
|---|---|---|---|---|---|
| **Probability of Varenicline Treatment** | Prior authorization | High (≥US$25) | 118,397 | 0.10 | 0.09, 0.12 |
| | | Low (<US$25) | 26,884 | 0.41 | 0.35, 0.48 |
| | Smoking cessation program | High (≥US$25) | 81,228 | 0.19 | 0.17, 0.21 |
| | | Low (<US$25) | 6,033 | 1.37 | 1.16, 1.63 |
| | No restriction | High (≥US$25) | 22,470 | 0.86 | 0.78, 0.96 |
| | | Low (<US$25) | 567,381 | 1.00 | N/A (reference) |

CI: confidence interval

### Medication Persistence

Results of logistic regression analysis, generalized linear models and Cox's proportional hazards models by exploring varenicline persistence are presented in Table 3. Within the no restriction cohort, patients with high out-of-pocket costs were 53% less likely to complete a full course of varenicline treatment than their counterparts with low out-of-pocket costs (OR=0.47; p=0.002). Compared with those with no access restrictions on varenicline and low out-of-pocket costs, the odds of a patient with a smoking cessation program requirement and low out-of-pocket costs completing a full course of varenicline treatment were 62% lower (OR=0.38; p=0.037), but the odds of completing a full course of varenicline were relatively higher (30%; OR=0.70; p=0.13) for patients with high out-of-pocket costs. Conversely, the odds of a patient with a prior authorization and low out-of-pocket cost requirement completing a full course of varenicline treatment were 157% higher vs. those with no access restrictions and a low out-of-pocket cost (OR=2.57; p<0.001). Among varenicline users with no access restrictions, the number of days' supply of varenicline was 16% lower in the high vs. the low out-of-pocket cohort (OR=0.84; p<0.001).

## DISCUSSION

Our results suggest that high out-of-pocket costs are associated with lower utilization of and persistence with varenicline treatment. Among patients with no access restrictions on the use of varenicline, the odds of varenicline treatment were 14% lower in the high out-of-pocket group than the low out-of-pocket group, suggesting that patients are influenced by the cost of varenicline and are less likely to undergo varenicline treatment if faced with higher costs. Our findings are consistent with those of a previous study that examined the impact of patient copayment on the utilization of varenicline[16] and reported that patients with a low varenicline copayment were significantly more likely to fill a prescription for any smoking medication than those who had a high copayment. Similarly, increased cost-sharing has been shown to result in lower rates of drug treatment and lower adherence across a variety of other medication classes.[12] Compared with poor adherence, good adherence to varenicline (≥80% of days taken) was associated with a two-fold increase in 6-month quit rates (52% vs. 25%).[27] In addition, adherence to drug therapy is known to improve health outcomes and reduce mortality.[28] This knowledge has implications in terms of the potentially negative impact that administration of high out-of-pocket could have on adherence to smoking cessation pharmacotherapy, and subsequently, clinical outcomes.

**Table 3.** Impact of Access Restrictions and Out-of-pocket (OOP) Cost on Varenicline Persistence

| Outcome | Access Level and Patient OOP Cost | | N | Odds Ratio | 95% CI |
|---|---|---|---|---|---|
| Probability of completing a course of varenicline | Prior authorization | High (≥US$25) | 213 | 0.74 | 0.44, 1.23 |
| | | Low (<US$25) | 103 | 2.57 | 1.70, 3.89 |
| | Smoking cessation program requirement | High (≥US$25) | 276 | 0.70 | 0.44, 1.11 |
| | | Low (<US$25) | 110 | 0.38 | 0.15, 0.95 |
| | No restriction | High (≥US$25) | 371 | 0.47 | 0.29, 0.76 |
| | | Low (<US$25) | 10,698 | 1.00 | N/A (reference) |
| **Outcome** | **Access Level and Patient OOP Cost** | | **N** | **Days Ratio** | **95% CI** |
| Days' supply of varenicline | Prior authorization | High (≥US$25) | 213 | 0.95 | 0.88, 1.04 |
| | | Low (<US$25) | 103 | 1.25 | 1.13, 1.39 |
| | Smoking cessation program requirement | High (≥US$25) | 276 | 0.92 | 0.86, 0.99 |
| | | Low (<US$25) | 110 | 0.97 | 0.87, 1.09 |
| | No restriction | High (≥US$25) | 371 | 0.84 | 0.79, 0.90 |
| | | Low (<US$25) | 10,698 | 1.00 | N/A (reference) |
| **Outcome** | **Access Level and Patient OOP Cost** | | **N** | **Days Ratio** | **95% CI** |
| Days to discontinuation of varenicline | Prior authorization | High (≥US$25) | 213 | 1.07 | 0.93, 1.23 |
| | | Low (<US$25) | 103 | 0.76 | 0.63, 0.90 |
| | Smoking cessation program requirement | High (≥US$25) | 276 | 1.11 | 0.99, 1.26 |
| | | Low (<US$25) | 110 | 1.07 | 0.89, 1.30 |
| | No restriction | High (≥US$25) | 371 | 1.25 | 1.12, 1.39 |
| | | Low (<US$25) | 10,698 | 1.00 | N/A (reference) |

CI: confidence interval; OOP: out-of-pocket

This study is a retrospective claims-based analysis, and as such, one of the main limitations is that patients were identified via administrative claims data as opposed to medical records, which could potentially lead to the misclassification of covariates and outcomes. Moreover, because information on smoking status is poorly coded in administrative claims, it was not possible to definitively determine the proportion of smokers in each cohort. Thus, some of the variation in utilization between cohorts may be related to differences in the proportion of smokers rather than out-of-pocket costs. The findings of this study are limited to data collected during a single year (2009); we were not able to assess whether there were changes in plan design or out-of-pocket costs from the previous year that might have impacted varenicline utilization in 2009. Additionally, because this study included individuals with commercial health coverage through their own or a family member's employer, the results may not be extrapolated to patient populations insured through other carriers such as Medicare or Medicaid. There may also have been systematic differences between the study cohorts that accounted for the detected differences in varenicline utilization or persistence (although this was partially controlled for using regression).

## CONCLUSIONS

This research suggests that high cost-sharing for varenicline results in lower utilization of and compliance with varenicline treatment. Given the demonstrated positive clinical and economic benefits associated with smoking cessation, and the data which show that varenicline is an effective treatment to assist smokers with quit attempts and to remain abstinent.[8,10,18] policy makers should consider whether restrictions and high out-of-pocket costs that may decrease utilization of the drug are beneficial.

Data from this study were presented at the *18th Annual Meeting of the Society for Research on Nicotine and Tobacco (SRNT)*, March 13–16, 2012; Houston, Texas, USA.

## ACKNOWLEDGMENTS

Data were analyzed by employees of Truven Health Analytics, which received funding from Pfizer Inc. for the conduct of this study. Editorial/medical writing support was provided by Alexandra Bound, PhD and Helen Jones, PhD, at Engage Scientific, and was funded by Pfizer Inc.

### Declaration of Competing Interests

This study was supported by Pfizer, Inc. AG, GM and KHZ are employees of and shareholders in Pfizer Inc. LM, KC and GL are employees of Truven Health Analytics, which received funding from Pfizer Inc. for the conduct of this study.
